# Insecticide resistance status and high frequency of *kdr* mutations in *Aedes aegypti* in Tegucigalpa, Honduras

**DOI:** 10.1186/s13071-025-06953-2

**Published:** 2025-08-01

**Authors:** Cindy Reyes-Perdomo, Denis Escobar, Luis Galo, Oscar Urrutia, Rita Lucrecia Vizcaino, Audrey Lenhart, Gustavo Fontecha

**Affiliations:** 1 Instituto de Investigaciones en Microbiología, Facultad de Ciencias, Universidad Nacional Autónoma de Honduras, Tegucigalpa, Honduras; 2 Unidad de Vigilancia de la Salud, Secretaría de Salud de Honduras, Tegucigalpa, Honduras; 3 Entomology Branch, Division of Parasitic Diseases and Malaria, US Centers for Disease Control and Prevention, Atlanta, GA USA

**Keywords:** *Aedes aegypti*, Dengue, Honduras, Insecticide resistance, Kdr

## Abstract

**Background:**

*Aedes aegypti* is the main vector of arboviruses in the Americas. Insecticide use remains the primary method for outbreak control, but prolonged application exerts selective pressure that promotes resistance. This study aimed to assess insecticide resistance and characterize key knockdown resistance (*kdr*) mutations in *Ae. aegypti* populations from the Central District of Honduras.

**Methods:**

Larvae were collected from four localities between May and June 2023. Susceptibility to four insecticides was evaluated via bioassays. Frequencies of the F1534C and V1016I *kdr* alleles and their haplotypes were determined, and sequencing of the *vgsc* gene was performed for further genotyping.

**Results:**

A total of 1592 *Ae. aegypti* females were phenotyped. All populations were resistant to permethrin and malathion, and two were resistant to deltamethrin; all were susceptible to bendiocarb. The 1534C mutant allele was fixed (1.0), and 1016I had an overall frequency of 0.89, with local variation from 0.48 to 1.0.

**Conclusions:**

Widespread resistance to commonly used insecticides was detected in *Ae. aegypti* from the Central District. High frequencies of *kdr* mutations underscore the need for continuous resistance monitoring to guide effective vector control strategies in Honduras.

**Graphical abstract:**

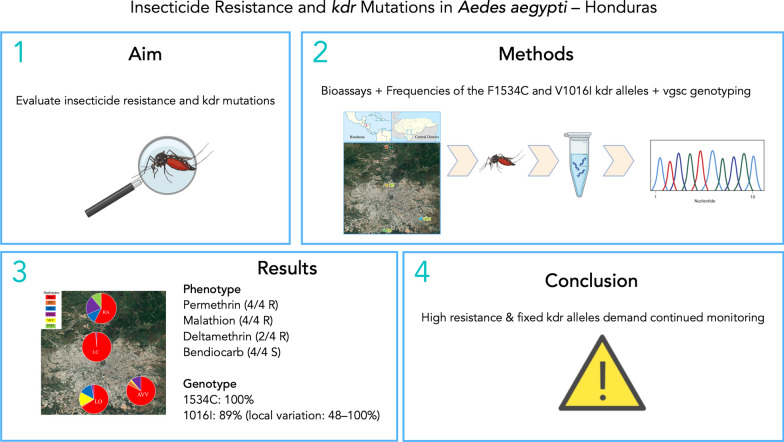

**Supplementary Information:**

The online version contains supplementary material available at 10.1186/s13071-025-06953-2.

## Background

*Aedes (Stegomyia) aegypti* is a major vector that has contributed to outbreaks of multiple arboviral diseases throughout the past century [1, 2]. Its vectorial capacity for new and re-emerging diseases has been projected to increase as a result of the impact of climate change, urbanization, and human movement patterns [3, 4]. Modeling analyses have provided indications that the distribution patterns of *Ae. aegypti* are anticipated to undergo significant transformations over the current century, as previously hostile habitats are transformed into more favorable environments [5, 6].

In recent decades, most tropical countries of the Americas have been severely impacted by recurring dengue epidemics, as well as the spread of new arboviruses such as chikungunya and Zika [7, 8]. The first cases of dengue in Honduras were documented during the 1978 epidemic, with a total of over 130,000 cases. In the following years, there were documented reports of epidemics in 1987, impacting around 29,000 individuals, as well as in 1989 and 1991. At that time, dengue occurred in large outbreaks, with a particular concentration in the two most densely inhabited cities, Tegucigalpa and San Pedro Sula [9]. Between 2015 and 2016, Honduras experienced the emergence of two additional arboviruses (chikungunya and Zika) transmitted by *Ae. aegypti*, in addition to ongoing dengue epidemics [10–12].

The main approach for controlling epidemic outbreaks of *Aedes*-borne arboviruses remains the control of the adult stage of the mosquito [13]. In Honduras, the Ministry of Health carries out vector control activities with insecticide fogging, often using a combination of permethrin plus a synergist (piperonyl butoxide) [14]. The recurrent use of insecticides often leads to the emergence of mosquito populations that are resistant to insecticides.

Phenotypic insecticide resistance in *Aedes aegypti* is associated with at least four documented biological mechanisms [15]. Among these, the most commonly reported are: (i) increased activity of detoxifying enzymes, representing a metabolic mechanism; and (ii) mutations in the target site of the insecticide within the mosquito’s nervous system, known as knockdown resistance (*kdr*) [16]. To date, 11 *kdr*-type mutations associated with pyrethroid resistance have been identified. Notably, combinations of mutations at positions 989, 1016, and 1534—such as V1016G/S989P, F1534C/V1016G, and F1534C/V1016I—have been widely documented in field populations [17–20].

Although insecticides are critical for controlling vectors, there is a lack of information regarding the status of resistance and the distribution of *kdr* mutations in *Ae. aegypti* in Central America. The available data from Latin America are primarily generated from Mexico and Brazil, with some additional reports from countries such as Colombia, Costa Rica, Cuba, Peru, and some Caribbean nations [21–26]. Nevertheless, significant knowledge gaps remain regarding the prevalence and magnitude of insecticide resistance in *Ae. aegypti* within the Central American region.

Thus, this study aimed to conduct a phenotypic assessment of insecticide resistance in *Ae. aegypti* populations collected in the Central District of Honduras. In addition, the study sought to characterize the frequency of *kdr* alleles in the populations.

## Methods

### Study site

*Aedes aegypti* were collected in the municipality of the Central District of Honduras. This region was selected because it includes the capital city of Tegucigalpa and reports the highest prevalence of dengue and other arboviruses in the country [27, 28]. The Central District is located between 900 and 1000 m above sea level and has an approximate population of 1.1 million inhabitants. There are two seasons, a dry season (from December to May) and a rainy season (from June to November). The average temperature ranges between 16 °C and 30 °C.

**Fig. 1:**
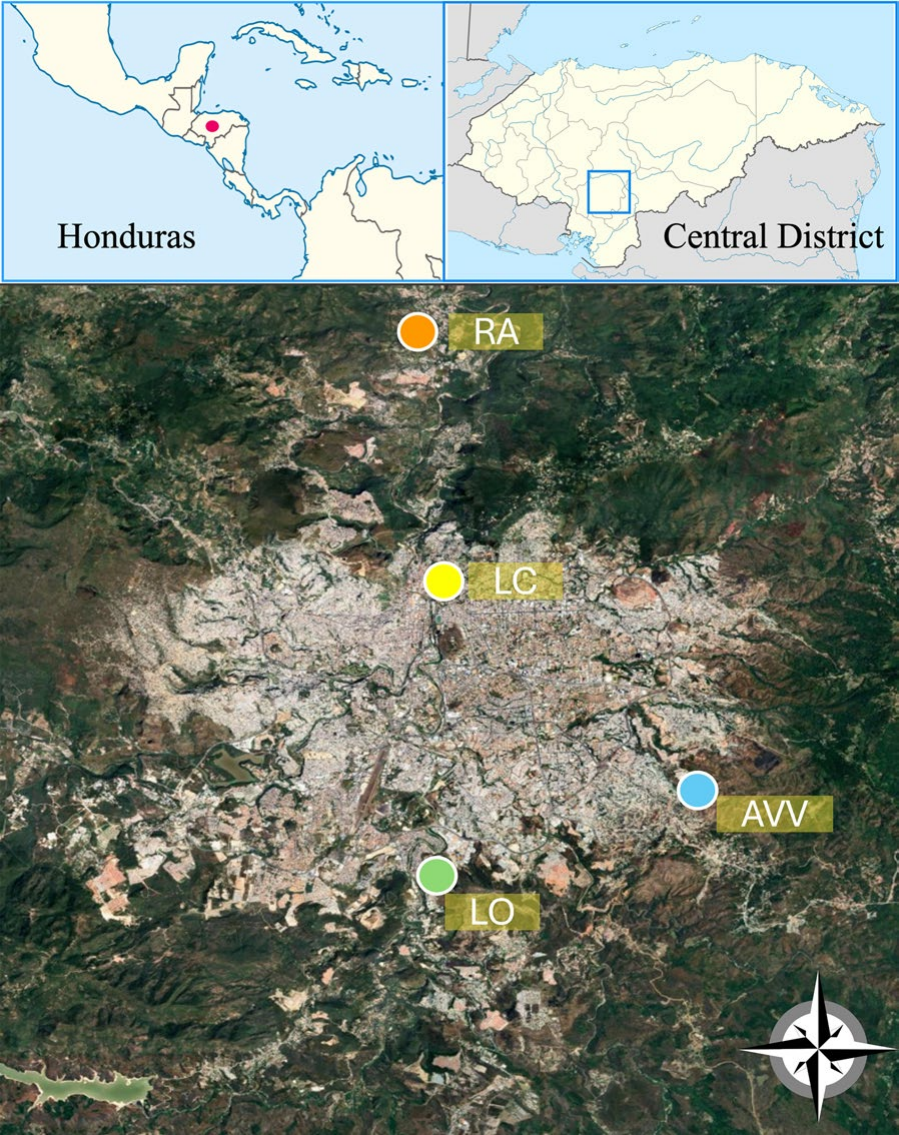
Map of the Central District of Honduras showing the four collection sites. Green dot: Loarque (LO); blue dot: Altos de Villa Vieja (AVV); yellow dot: La Concordia (LC); orange dot: Río Abajo (RA)

Four neighborhoods were selected to collect larvae: Loarque (LO; 14.041576−87.211082), La Concordia (LC;14.112068−87.208535), Altos de Villa Vieja (AVV; 14.059852−87.162463), and Río Abajo (RA; 14.173899−87.211295) (Fig. 1). These neighborhoods were selected because they were reported as those with the highest routine use of insecticide spraying by the Ministry of Health of Honduras.

### Collection and rearing of mosquitoes

The study involved visiting between five and seven locations inside each of the four chosen neighborhoods that exhibited clear evidence of water accumulation, including sinks, pans, and automobile tires. Mosquito larvae and pupae were collected at each site [29]. The live larvae and pupae were transferred in plastic containers to the Medical Entomology Laboratory of the Universidad Nacional Autonoma de Honduras (UNAH).

Larvae and pupae obtained from the field (F0 generation) were reared to adults under standard insectary settings. Each group of larvae collected from neighborhoods was placed separately in trays (28 cm × 18 cm × 6 cm) with 1500 mL of distilled water. The larvae were fed daily with fish food (Biomaa, Super Flakes, Mexico) and maintained at a relative humidity of 70–80%, a temperature range of 25–27 °C, and a light and dark cycle of 12 h each, until they reached maturity [30–32]. Emerging adult mosquitoes were identified using taxonomic keys [33]. All individuals identified as *Aedes aegypti* were separated by population and housed in individual cages, where they were allowed to intermate. Blood feeding was performed using a Hemotek blood feeding system (Blackburn, UK). Subsequently, 3 days post-feeding, ovitraps were placed to collect eggs, which were reared to the F1 generation, following previously described protocols [31]. All mosquitoes were maintained on a 10% sugar solution until their use in insecticide susceptibility bioassays.

### Phenotypic bioassays

Susceptibility bioassays were performed following the procedures described by the Centers for Disease Control and Prevention (CDC) bottle bioassay method [34]. Briefly, the diagnostic concentrations of four insecticides were prepared from technical grade active ingredients (Chem Services, West Chester, PA, USA): two pyrethroids (deltamethrin, 10 µg/mL and permethrin, 15 µg/mL); a carbamate (bendiocarb, 12.5 µg/mL), and an organophosphate (malathion, 50 µg/mL). The bioassays were performed using 3–5-day-old non-blood-fed adult female *Ae. aegypti*. Between 20 and 25 mosquitoes were placed into 250 mL Wheaton bottles previously impregnated with the diagnostic concentration of insecticide and kept under observation for 30 min. The number of knocked down individuals was recorded at 0, 15, and 30 min of exposure. The diagnostic time to determine the phenotype was 30 min, according to what is described by the CDC method [34]. Subsequently, all exposed mosquitoes were transferred to holding containers with 10% sugar solution to evaluate mortality after 24 h (as a proxy for recovery). After recording the mortality rate at 24 h, both susceptible and resistant individuals were separated and kept at −20 °C until molecular analyses were performed.

### DNA extraction and genotyping of positions 1534 and 1016 of the *vgsc* gene

For the detection of *kdr* mutations, a sample of 285 individuals from the F1 generation, who were phenotyped in deltamethrin and permethrin bioassays, was randomly chosen from the four study locations: 70 individuals (25%) from AVV, 80 (28%) from LC, 74 (26%) from LO, and 61 (21%) from RA. Individuals of both resistant (*n* = 135) and susceptible (*n* = 150) phenotypes were selected. DNA was extracted separately from each mosquito using the ReliaPrep^™^ Blood gDNA Miniprep System (Promega, Madison, Wisconsin, USA), according to the manufacturer’s instructions.

Genotyping of positions 1534 and 1016 was carried out using allele-specific polymerase chain reaction (AS-PCR) according to the primers and protocols described by Yanola et al. [11] and the primers modified by Contreras-Perera et al. [35] (Table 1). The possible genotypes for position 1534 were F1534F (homozygous wild type), F1534C (heterozygous mutant), and C1534C (homozygous mutant). For position 1016, the possible genotypes were V1016V (homozygous wild type), V1016I (heterozygous mutant), and I1016I (homozygous mutant). For both loci, the presence of heterozygotes was registered by observing the presence of double bands according to the base pair sizes previously reported. Briefly, PCR reactions to analyze position 1534 were carried out in a final volume of 20 μL, including 10 μL of Taq Master Mix 2X (Promega, Madison, Wisconsin, USA), 0.8 μL of each primer (10 μM) (Table 1), 1 μL of DNA, and 6.6 μL of nuclease-free water (NFW). The amplification conditions were as follows: 95 °C for 5 min, 35 cycles of 95 °C for 1 min, 57 °C for 1 min, and 72 °C for 1 min, with a final extension step at 72 °C for 4 min.

**Table 1:** Sequences of the primers and PCR conditions used for genotyping positions 1534 and 1016 of the *vgsc* gene

Target	Primer	Primer sequence	Annealing temperature (°C)	PCR product size (base pair (bp))
IIS5-6	C1534fw	5′-GCG GGC AGG GCG GCG GGG GCG GGG CCT CTA CTT TGT GTT CTT CAT CAT GTG-3′	57	93 113
	F1534fw	5′-GCG GGC TCT ACT TTG TGT TCT TCA TCA TAT T-3′		
	F1534R	5′-TCT GCT CGT TGA AGT TGT CGA T-3′		
IIIS5-6	Val1016fw	5′-GCG GGC AGG GCG GCG GGG GCG GGG CCA CAA ATT GTT TCC CAC CCG CAC CGG-3′	60	102 82
	Ile1016f	5′-GCG GGC ACA AAT TGT TTC CCA CCC GCA CTG A-3′		
	Ile1016r	5′-TGA TGA ACC SGA ATT GGA CAA AAG C-3′		
IIS5-6	IIS5-6F	5′-ATC GCT TCC CGG ACA AAG AC-3′	50	562–Intron A 579–Intron B
	IIS5-6R	5′-GTT GGC GAT GTT CGA CTT GA-3′		
IIIS5-6	AaNa31F	5′-GAC TCG CGG GAG GTA AGT T-3′	59	512
	AaNa31R	5′-CCG TCT GCT TGT AGT GAT CG-3′		

For position 1016, the PCR reaction was carried out in a volume of 25 μL, with 12.5 μL of Taq Master Mix 2X (Promega, Madison, Wisconsin, USA), 1 μL of each primer (10 μM) (Table 1), 4 μL of DNA, and 5.5 μL of NFW. The amplification conditions were as follows: 95 °C for 5 min, 29 cycles of 95 °C for 1 min, 60 °C for 1 min, and 72 °C for 1 min, with a final extension step at 72 °C for 10 min. The amplification products were separated by electrophoresis on a 3% agarose gel and visualized with ethidium bromide under ultraviolet (UV) light.

### Genotyping of positions 989, 1011, and 1520 of the *vgsc* gene

To detect additional mutations at 989, 1011, and 1520 positions at the voltage-gated sodium channel gene, a partial region of the IIS5-6 and IIIS5-6 segments was amplified using specific primers reported elsewhere (Table 1) [36, 37]. To identify additional *kdr* mutations associated with resistance to pyrethroid insecticides, the DNA extracted from 60 individuals was selected: 28 individuals resistant to the pyrethroids deltamethrin (*n* = 9) and permethrin (*n* = 19), and 32 individuals susceptible to deltamethrin (*n* = 17) and permethrin (*n* = 15). Briefly, the IIS5-6 fragment was amplified in a final volume of 50 μL, including 25 μL of Taq Master Mix 2X (Promega, Madison, Wisconsin, USA), 2 μL of each primer (10 μM) (Table 1), 4 μL of DNA, and 17 μL of NFW. The amplification conditions were as follows: 95 °C for 2 min, 40 cycles of 94 °C for 30 s, 50 °C for 30 s, and 72 °C for 1 min, with a final extension at 72 °C for 10 min.

PCR reactions for the IIIS5-6 fragment were carried out in a final volume of 50 μL, including 25 μL of Taq Master Mix 2X (Promega, Madison, Wisconsin, USA), 2.5 μL of each primer (10 μM) (Table 1), 2 μL of DNA, and 18 μL of NFW, under the following conditions: 95 °C for 5 min, 35 cycles of 94 °C for 30 s, 59 °C for 30 s, and 72 °C for 1 min, with a final extension at 72 °C for 10 min. The amplified products were separated on 1% agarose gels and visualized with ethidium bromide under UV light. The amplified products were sequenced by Psomagen^®^ (https://www.psomagen.com) using internal primers: 508R: 5′-TTG TTC GTT TCG TTG TCG GC-3′ for the IIS6 fragment and 71F: 5′-GTC CTC GAT CCT TCC AGG TG-3′ for the IIIS6 fragment. The sequences obtained were curated and analyzed with Geneious Prime 2024.0.2 software (Dotmatics, Auckland, New Zealand). Each sequence was aligned and compared with a reference sequence of *Ae. aegypti* (accession no. AAEL023266) available from VectorBase (https://vectorbase.org/vectorbase) [38] and other sequences available in the National Center for Biotechnology Information (NCBI) (accession nos. KY626180.1, KY626197.1, KY046222.1, XM_021852349.1) previously reported with mutations at positions 989, 1011, and 1016.

### Statistical analysis

Mortality was defined as the percentage of individuals knocked down at the diagnostic time. To categorize the susceptibility status of the populations, the ranges described by the CDC were followed: 98–100% mortality indicated susceptibility of the population, 90–97% indicated the development of resistance, and < 90% indicated resistance. Both allele frequencies and haplotype frequencies were calculated for each population and phenotype [39]. Wright’s inbreeding coefficient (FIS) was calculated to evaluate the population structure, where possible, considering the loci of interest using the formula: FIS = 1 − (Ho/He), where Ho is the number of observed heterozygotes, and He was calculated as follows: He = 2np (1 − *P*), where *n* is the sample size [40, 41]. The chi-squared test was calculated in R software (version 2024.04.1; https://www.R-project.org/) under the package “stats—> “*chisq.test”* to determine a significant association between phenotypic susceptibility and the F1534C, V1016I, and V1016V alleles (*P* < 0.05) [42].

## Results

### Bioassays

**Fig. 2:**
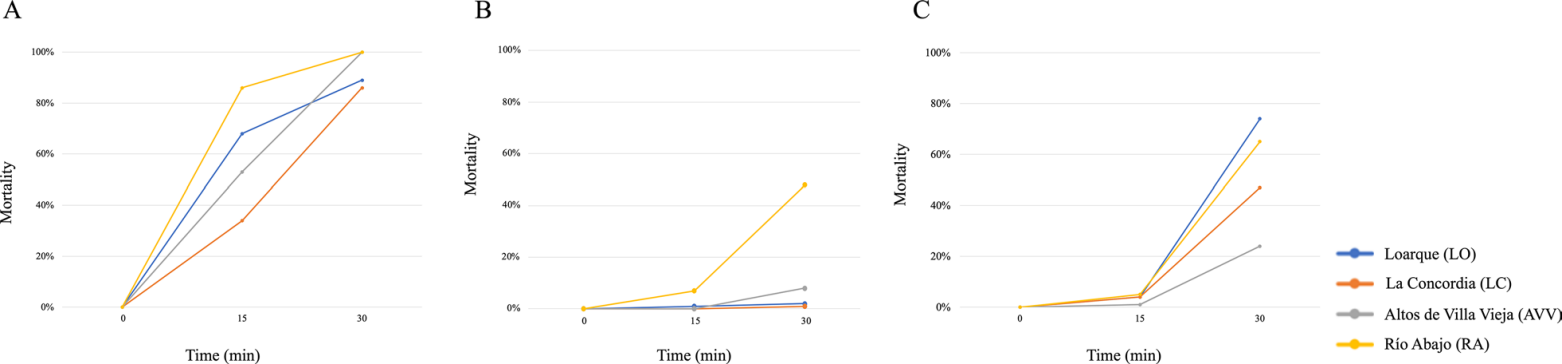
Bioassay mortality for each population at the diagnostic time of 30 min. (**A**) deltamethrin; (**B**) permethrin; (**C**) malathion

A total of 1592 *Ae. aegypti* females of the F1 generation were analyzed in bioassays. Four sets of bioassays were carried out per location, one per insecticide. All populations were resistant to permethrin and malathion. For permethrin, the mortality ranged between 1% and 48%, with LC showing the lowest mortality (1%) and RA showing the highest (48%) (Fig. 2). For malathion, mortality ranged between 24% (AVV) and 74% (LO). Mortality rates for deltamethrin ranged between 86% (LC) and 100% (RA and AVV). All populations showed 100% mortality at the diagnostic time for bendiocarb.

Of the four locations evaluated, only the LC population showed changes in mortality for both pyrethroids 24 h after exposure, with a recovery rate of 15% and 1% for deltamethrin and permethrin, respectively. (Additional File 1: Additional Table 1).

### *kdr* genotypes and haplotypes for positions 1016 and 1534

Out of the 285 individuals tested, 275 (96.5%) were successfully genotyped for at least one of the two loci, *n* = 275 for position 1534 and *n* = 273 for position 1016. No permethrin-susceptible phenotypes were detected from LC, nor deltamethrin-resistant phenotypes from AVV or RA, so these were not included in the genetic analyses. The allele frequencies by insecticide and phenotype are summarized in Table 2.

**Table 2:** Frequency of 1534 and 1016 *kdr* alleles per site

Neighborhood	*n*	CC	FC	FF	Frequency (Freq) C	Freq FC	*n*	II	VI	VV	Freq I	Freq VI
AVV	66	62	4	0	1.00	0.06	67	55	4	8	0.88	0.06
LC	78	78	0	0	1.00	0	78	75	2	1	0.99	0.03
LO	71	60	11	0	1.00	0.15	73	46	24	3	0.96	0.33
RA	60	47	13	0	1.00	0.22	55	30	8	17	0.69	0.15

*CC* homozygous mutant (C/C), *FC* heterozygous (F/C), *FF* homozygous wild-type, *II* homozygous mutant (I/I), *VI* heterozygous, *VV* homozygous wild-type

Genotyping analyses revealed the presence of individuals with the F1534C mutation in its homozygous mutant (C/C) and heterozygous (F/C) variants. In these populations, no individual was found with the wild variant (F/F). In contrast, the three genotypes were observed at position 1016 (V/V, V/I, and I/I). The double homozygous mutant haplotype (1534 C/C, 1016 I/I) was detected in 196 of 285 individuals analyzed (68.8%). The mosquitoes from LC accounted for 38% of the individuals with the double homozygous mutant haplotype, closely followed by the population from AVV. The allele frequencies for the mutant alleles were 1.0 and 0.89 for F1534C and V1016I, respectively.

Among the four populations evaluated, the 1534C allele exhibited a frequency of 1.0 across all locations. Although homozygous mutant allele was highly frequent, heterozygotes were observed in three out of the four populations studied: AVV (0.06), RA (0.22), and LO (0.15); in the latter, particularly, only five individuals (0.07) resistant to permethrin showed this genotype. On the contrary, the frequency of the V1016I resistant alleles showed variability between locations, ranging from 0.69 in RA to 0.99 in LC populations. Heterozygotes alleles (V/I) were observed in the four populations; of those, the highest frequency was observed in LO (0.33). Wright’s inbreeding coefficient (FIS) calculations showed a low relationship between the populations analyzed at position 1016, with a slight excess of heterozygosity in the RA and AVV populations (Additional File 1: Supplementary Table 2).

**Fig. 3:**
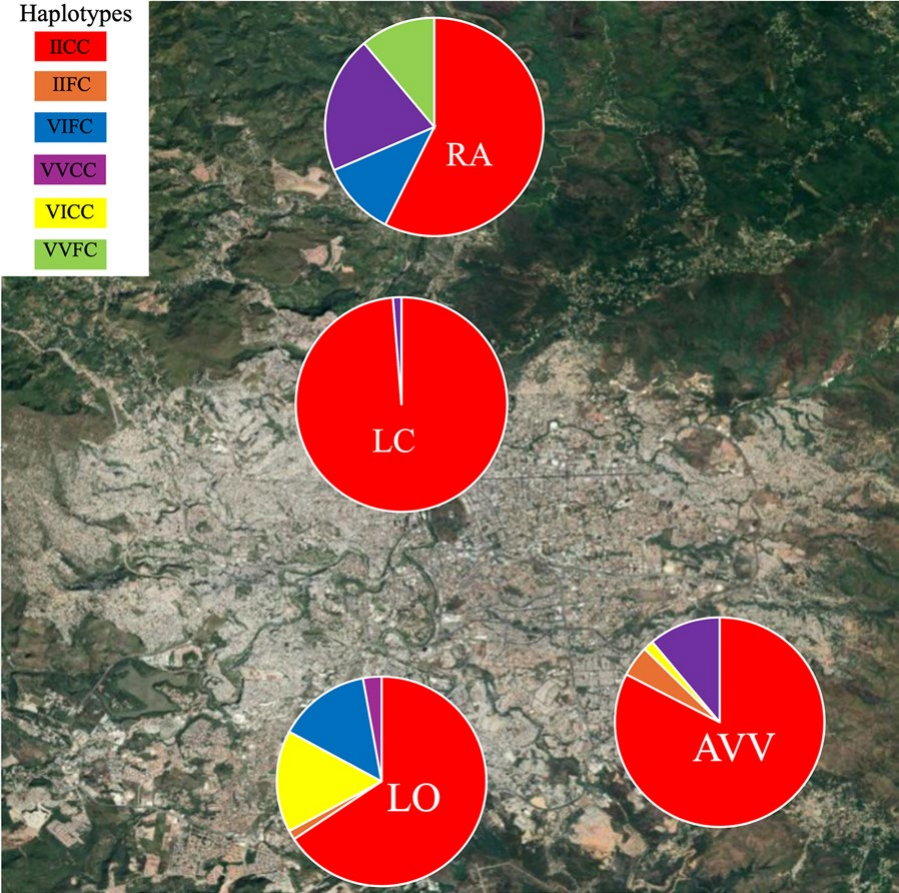
Haplotype distribution within each locality. Loarque (LO); Altos de Villa Vieja (AVV); La Concordia (LC); Río Abajo (RA)

On the basis of the observed genotypes, the haplotypes (1016/1534) were calculated by locality (Fig. 3). Six haplotypes were observed: R1R2 (II/CC), R1H2 (II/FC), H1R2 (VI/CC), H1H2 (VI/FC), S1R2 (VV/CC), S1H2 (VV/FC). Haplotype R1R2 (II/CC) was the most frequent haplotype in all localities, with the highest frequency observed in LC (0.99), followed by AVV (0.81). However, the S1H2 haplotype (VV/FC) had an overall frequency of 0.03 and was reported only in the AVV and RA localities.

When combining results from phenotype and haplotype, only populations from two sites, LO and LC, were resistant to both deltamethrin and permethrin. Both populations showed a high proportion of R1R2 haplotype (˃ 0.6) and nearly no S1R2 (< 0.03). These results contrast with AVV, whose population was susceptible to deltamethrin and where R1R2 was present at a frequency of 0.81, but with a frequency of 0.11 of the S1R2 haplotype. As F1534C heterozygotes were observed mostly in susceptible individuals, chi-squared tests were used to evaluate an association between the F1534C allele and the susceptible phenotype, and the V1016I allele underwent the same analysis; an additional test were carried out to test association between V1016V allele and susceptible phenotype. This revealed a significant association between heterozygotes and the susceptible phenotype, both for F1534C (*χ*^2^ = 5.6209; *df* = 1; *P* = 0.01775) and V1016I (*χ*^2^ = 5.9001; *df* = 1; *P* = 0.01514). Finally, V1016V showed a strong association with susceptible phenotype (*χ*^2^ = 20.881; *df* = 1; *P* = < 0.00001).

### Genotyping of positions 989, 1011, and 1520 on the *voltage-gated sodium channel* (*vgsc*) gene

Sequence analyses for the IIS6 and IIIS6 segments showed that all individuals presented the wild genotypes at positions 1520 (ACC), 989 (ATC), and 1011 (ATA). In addition, the sequencing data confirmed the genotypes identified by AS-PCR at positions 1016 and 1534.

## Discussion

The long-term effectiveness of vector control interventions is increasingly threatened by the emergence and spread of insecticide resistance [43]. Monitoring insecticide susceptibility and characterizing underlying resistance mechanisms are essential for informing and optimizing vector control strategies [44, 45]. This study contributes valuable data on the current status of insecticide resistance and reveals a high frequency of *kdr* alleles in *Aedes aegypti* populations from four localities in the vicinity of Tegucigalpa, Honduras.

There have been numerous reports of *Ae. aegypti* populations exhibiting resistance to multiple insecticides around the world. However, information on insecticide susceptibility in Honduras and the Central American region is still scarce [21]. The results of the present study showed populations resistant to two pyrethroids and the organophosphate malathion but full susceptibility to the carbamate bendiocarb. The Ministry of Health of Honduras has used commercial formulations with pyrethroids for control interventions for more than two decades, which has probably led to the selection of resistance in populations of *Ae. aegypti*. This phenomenon has been reported elsewhere in the Americas, such as in Mexico and Brazil, where populations highly resistant to pyrethroids have been observed after consecutive periods of insecticide application [16, 40, 46, 47].

The presence of two mosquito populations (RA and AVV) resistant to permethrin (a type I pyrethroid) but susceptible to deltamethrin (a type II pyrethroid) suggests that resistance to one pyrethroid does not always result in cross-resistance across the pyrethroid class. In the Americas, there are reports with similar results, showing lower mortality to permethrin compared with deltamethrin [22, 48]. The LC and LO populations showed resistance to both pyrethroids. These populations came from primarily residential areas, which may be subject to additional selection pressure by insecticides for domestic use, a phenomenon also observed in some areas of Brazil and Mexico [49, 50].

In Honduras, organophosphate insecticides were replaced by pyrethroids in the late 1990s to control *Ae. aegypti*. However, the results reported here reveal that populations continue to show resistance to the organophosphate malathion. A study in Jamaica in 2015 observed that populations of *Ae. aegypti* showed heterogeneous results to malathion in five populations, with mortalities between 84% and 90%, after a rotation from malathion to permethrin in 2014 during a chikungunya outbreak [25]. In addition, a study published by Rubio-Palis et al. in 2023 found that, despite reducing the use of organophosphates since 2010, four populations of *Ae. aegypti* continued to present resistance to malathion in Venezuela in 2020, demonstrating that despite the absence of pressure, phenotypic resistance was maintained [25, 51].

In addition to the phenotypic resistance findings, this study identified high frequencies of both the F1534C and V1016I *kdr* mutations. These alleles are widely distributed in *Aedes aegypti* populations globally, with F1534C being the most commonly reported mutation associated with reduced susceptibility to pyrethroids [16, 52]. Although the 1534C mutation seems almost fixed in the populations analyzed, association tests revealed an association between the heterozygous individuals from two neighborhoods and the susceptible phenotype, a pattern also observed for the V1016I allele. Reports from Brazil and Mexico have demonstrated the rapid evolution and distribution of *kdr* mutations in *Ae. aegypti*, revealing that these mutations are capable of arising independently and fixing quickly in local populations, probably driven by highly local selection factors [46, 47, 53–55].

Notably, this study detected F1534C and V1016I mutant alleles in both susceptible and resistant *Ae. aegypti* individuals. A previous study in Venezuela similarly reported the presence of the F1534C mutation in populations susceptible to cypermethrin, despite the mutation being fixed, highlighting the complexity of resistance, which likely involves multiple interacting mechanisms [48]. To further elucidate the mechanisms contributing to the resistance in the populations analyzed here, future investigations should include the characterization of key detoxification enzyme families, such as mixed-function oxidases (MFOs) and glutathione S-transferases (GSTs), which are known to metabolize insecticides and may play a significant role in the observed resistant phenotypes [56, 57].

The combination of multiple mutant alleles on the mosquito *vgsc* gene can result in haplotypes that are associated with high levels of resistance to pyrethroids [58, 59]. Reports from Brazil have demonstrated the wide distribution of the 1534/1016 homozygous mutant haplotype (C/C, I/I). A recent national survey carried out in 123 municipalities in Brazil showed that this haplotype was the second most frequent nationally (24%). In Florida, USA, another investigation examined populations of *Ae. aegypti* in 62 locations, revealing a significant occurrence of the double-mutant combination [60]. This double-mutant haplotype was also the most frequent in the four populations we studied from the Central District of Honduras. Although the haplotypes were not predictive of resistance phenotype (likely due to the contributions of additional resistance mechanisms), the variation in the frequencies of *kdr* haplotypes across the neighborhoods suggests a highly focal variation in selection that is occurring at the local level.

Additional *kdr* mutations in the *vgsc* gene, such as T1520I, S989P, and I1011V/M, have been primarily reported in *Ae. aegypti* populations from Asia [16, 37]. Although this study did not detect nonsynonymous mutations at positions 1520, 989, or 1011, the continued use of pyrethroids, along with the potential introduction of mosquitoes from other regions, may facilitate the emergence of these or other resistance-associated mutations in the future [17].

## Conclusions

This study revealed phenotypic resistance to permethrin and malathion in *Ae. aegypti* populations across the study sites, and variable levels of deltamethrin susceptibility. The *kdr* alleles F1534C and V1016I were detected at high frequencies in *Ae. aegypti*, together with a predominant double-mutant haplotype (C/C, I/I). These results can inform vector control decision-making and can be considered as a baseline for prospective monitoring of resistance trends.

## Supplementary Information

Additional file 1.

## Data Availability

Data supporting the main conclusions of this study are included in the manuscript.

## Abbreviations

CDC: Centers for Disease Control and Prevention
*Kdr*: Knockdown resistance
*vgsc*: Voltage-gated sodium channel
