# Supplementary material for: Insecticide resistance status and high frequency of kdr mutations in Aedes aegypti in Tegucigalpa, Honduras
Source: Parasit Vectors. 2025 Aug 1;18:321. doi: 10.1186/s13071-025-06953-2 (PMC12317443; doi:10.1186/s13071-025-06953-2)
Supplement: Supplementary file 1 — Additional file 1. [file 13071_2025_6953_MOESM1_ESM.docx]

**Additional files**

**Additional Table 1**. Mosquito mortality percentages after 24 h exposure, by insecticide and location

| **Insecticide** | **Neighborhood** | ***n*** | **Mortality at 30 min (%)** | **Mortality at 24 hrs (%)** |
| --- | --- | --- | --- | --- |
| **Deltamethrin** | LO | 94 | 89 | 96 |
|  | LC | 100 | 86 | **71*** |
|  | AVV | 100 | 100 | 100 |
|  | RA | 100 | 100 | 100 |
| **Permethrin** | LO | 98 | 2 | 12 |
|  | LC | 100 | 1 | **0*** |
|  | AVV | 100 | 8 | 21 |
|  | RA | 100 | 48 | 85 |
| **Malathion** | LO | 100 | 74 | 99 |
|  | LC | 100 | 24 | 100 |
|  | AVV | 100 | 47 | 100 |
|  | RA | 100 | 65 | 100 |
| **Bendiocarb** | LO | 100 | 100 | 100 |
|  | LC | 100 | 100 | 100 |
|  | AVV | 100 | 100 | 100 |
|  | RA | 100 | 100 | 100 |

* Populations where mortality changes were observed.

**Additional Table 2**. Wright's inbreeding coefficient (FIS) for position 1016 according to each neighborhood

| **Neighborhood** | ***n*** | **II** | **VI** | **VV** | **Freq I** | ***He*** | ***Fis*** |
| --- | --- | --- | --- | --- | --- | --- | --- |
| AVV | 67 | 55 | 4 | 8 | 0.88 | 14.09 | 0.716 |
| LC | 78 | 75 | 2 | 1 | 0.99 | 1.97 | -0.013 |
| LO | 73 | 46 | 24 | 3 | 0.96 | 5.75 | -3.171 |
| RA | 55 | 30 | 8 | 17 | 0.69 | 23.49 | 0.659 |
